# Did the COVID-19 Pandemic Change the Anaesthesia Preferences of Pregnant Women for Caesarean Section?

**DOI:** 10.5152/TJAR.2022.22058

**Published:** 2022-12-01

**Authors:** Muhammet Korkusuz, Tayfun Et

**Affiliations:** 1 Department of Anaesthesiology and Reanimation, Karaman Training and Research Hospital, Karaman, Turkey

**Keywords:** Caesarean section, COVID-19, obstetric anaesthesia, pandemic, regional anaesthesia

## Abstract

**Objective:**

The COVID-19 disease has become a new cause of anxiety for pregnant women regarding caesarean sections. The purpose of the present study was to investigate the anaesthesia preferences of pregnant women who preferred general anaesthesia in their previous caesarean sections during the COVID-19 pandemic.

**Methods:**

A total of 140 pregnant women between the ages of 18 and 45, who had undergone elective caesarean section under general anaesthesia, were included in the study. Amsterdam Preoperative Anxiety and Information Scale and Beck Anxiety Inventory were applied. The primary cause of anxiety and anaesthesia preference was asked and recorded.

**Results:**

It was found that 50.7% (71/140) of the pregnant women who preferred general anaesthesia in their previous caesarean sections preferred regional anaesthesia during the COVID-19 pandemic. It was also found that patients with primary anxiety because of COVID-19 contagion preferred regional anaesthesia more (36/55), and the primary reason for anxiety in these patients was COVID-19 contamination anxiety (36/71).

**Conclusions:**

Informing the patients about the pandemic precautions in the surgery room and in the hospital during the pandemic, as well as routine information in the preoperative anaesthesia appointment, may be effective in the choice of regional anaesthesia, which is considered to have a low risk of transmission.

**Main Points**

- 50.7% (71/140) of the pregnant women who preferred general anaesthesia in their previous caesarean sections preferred regional anaesthesia in their caesarean sections during the COVID-19 pandemic.
- The primary cause of anxiety in the majority of patients who prefer regional anaesthesia is the concern of contamination with COVID-19 (36/71).
- Patients who have primary anxiety due to COVID-19 contamination during hospitalization and caesarean section mostly prefer regional anaesthesia (36/55).

## Introduction

Caesarean section is a common surgical procedure increasing in obstetric patients in parallel with the increasing population. General anaesthesia and regional anaesthesia are anaesthesia methods that are applied in caesarean delivery. Regional anaesthesia is the most preferred method compared to general anaesthesia by patients and anaesthesiologists.

Anxiety is a psychological reaction that the individual develops against situations in which she does not feel safe. Anaesthesia concern, risk of death, disability, fear of pain, loss of control over the body, loss of sexual competence, and loss of workability are among the causes of anxiety in patients who are scheduled for surgical intervention.^1,2^ There are publications in the literature reporting that anxiety is more common in the surgery of obstetric patients than in other surgical procedures.^3,4^

Previous studies show that patients who cannot cope with the additional difficulties of being awake during surgery prefer general anaesthesia.^5,6^ It was observed that the frequency of anxiety is high (72.7%) in patients who are scheduled for elective caesarean section, and general anaesthesia is preferred in patients with anxiety.^7^

The fact that people have to undergo surgery for any procedure, especially during COVID-19, which is frequently transmitted through the respiratory tract and causes a respiratory tract infection, is an additional reason for anxiety because of the risk of infection. In this process, the recommendations of anaesthesiologists have increasingly continued to be regional anaesthesia for caesarean sections to protect patients as much as possible from the respiratory-borne COVID-19 disease. Although pregnant women mostly prefer regional anaesthesia, it is a matter of curiosity whether pregnant women who preferred general anaesthesia in their previous caesarean sections because of various anxieties changed their preferences during the pandemic period. For this reason, the hypothesis of this study was based on the assumption that pregnant women who preferred general anaesthesia in their previous caesarean sections could change their anaesthesia preferences in their caesarean sections during the pandemic period because of less exposure to the risk of infection transmission. The secondary purpose was to evaluate the preoperative anxiety factors of patients who preferred general anaesthesia or regional anaesthesia along with the anxiety of COVID-19 infection, which is an emerging cause of anxiety.

## Methods

The present study was conducted in Karaman Training and Research Hospital between July 1, 2021, and September 30, 2021, after the ethics committee approval was received from Karamanoglu Mehmetbey University, School of Medicine Ethical Committee.

The patients who were aged between 18 and 45, who voluntarily preferred general anaesthesia in the previous caesarean sections, who had undergone caesarean sections under general anaesthesia, who were scheduled for elective caesarean sections in the American Society of Anaesthesiologists I-III risk group, and who agreed to participate in the study were included in the present study. Those with psychiatric disorders, and therefore used drugs, who had bad obstetric history, who had a complicated pregnancy or congenital fetal anomaly were not included in the study.

The patients were evaluated and informed by the researcher in the preoperative anaesthesia interview conducted 1 day before the scheduled caesarean section. Information was given on the risks of transmission of COVID-19 disease, as well as the routine preoperative anaesthesia interview. COVID-19 measures taken in the hospital and surgery room were explained; 1 hour of evaluation time was given to ask about the anaesthesia preference and to apply the questionnaire. After the consent was obtained for anaesthesia, they were asked to indicate their preference for anaesthesia and to fill out the questionnaire.

The questionnaire consisted of the following 3 parts: demographic data form, Amsterdam Preoperative Anxiety and Information Scale (APAIS), and Beck Anxiety Inventory (BAI).

### Demographic Data Form

The form consisted of 7 questions and was prepared by the researchers in line with the literature data, including the descriptive characteristics of the patients, the reasons that might affect the anaesthesia preferences, reasons for preference in the preoperative period, and the variables to determine the causes of preoperative anxiety.

### Amsterdam Preoperative Anxiety and Information Scale

The scale is one of the tests used in the evaluation of preoperative anxiety. The Moermann group in the Netherlands developed APAIS in 1996. In this test, the source of concern is divided into 3 as anxiety about surgery, anxiety about anaesthesia, and anxiety caused by lack of knowledge. It includes 6 statements for these 3 sources to evaluate anxiety. Each statement is given a numerical value based on a 5-point Likert design according to severity to objectify the questionnaire. These values range between 1 and 5; 1 = none, 2 = mild, 3 = moderate, 4 = severe, 5 = extreme severity. Anaesthesia anxiety is evaluated by the answers given to questions 1 and 2, surgical anxiety with questions 4 and 5, and the total anxiety score by summing both. The statements that refer to the desire to obtain information on anaesthesia and surgery are in questions 3 and 6. The lowest score is 6, and the highest score is 30.^8^ It was used by Aykent et al^9^ for the first time in our country after adapting it into Turkish.

### Beck Anxiety Inventory

Beck Anxiety Inventory is rated on a 4-point Likert-type scale from 0 to 3 with overall scores ranging between 0 and 63. If the BAI score is within the range of 0-9, it is interpreted as minimal or no anxiety. A range of 10-18 points indicates mild anxiety, 19-29 indicates moderate anxiety, and 30-63 indicates severe anxiety. Beck Anxiety Inventory was developed to differentiate between anxiety and depression.^10^ The Turkish version of this questionnaire was adapted by Ulusoy et al.^11^ The scale is used to obtain an anxiety assessment independent of depression.

### Statistical Analysis

A power analysis was made by predicting that 40% of the patients who applied for elective caesarean section and received general anaesthesia for the same reason would prefer regional anaesthesia during the pandemic process after being informed about the risk of transmission of COVID-19. The priori sample size was calculated with a 5% margin of error and 95% CI with an anticipated effect size of 0.5 and 140 patients were included in the study. 

The frequencies, rates, mean, and standard deviations of the participants in terms of different variables were presented as descriptive statistics, numbers, and percentage values for categorical variables and mean and standard deviation values for continuous variables in the tables. Whether the distributions of the study variables met the normality assumption was evaluated with the Skewness and Kurtosis levels and histograms. Evaluation results show that the study variables met the normality assumption. The independent groups *t*-test and one-way analysis of variance were used to compare the mean scores, and mean ± standard deviation values were reported. The chi-square analysis was made and cross tables were added to examine whether there were significant differences between the distribution rates of the groups into categories in categorical data. The significance level for all analysis results was taken as <.05. The data analysis was made by using the Statistical Package for the Social Sciences 25 (IBM Corp.; Armonk, NY, USA) program.

## Results

The sample of this study consisted of 140 pregnant women who preferred general anaesthesia in their previous elective caesarean sections and who were interviewed for preoperative anaesthesia 1 day before the surgery for repeated caesarean section and accepted to apply the questionnaire.

Demographic profile of the participants is presented in Table 1.

According to our survey results, those who chose the risk of COVID-19 transmission as a primary concern rather than surgery or anaesthesia were significantly more likely to prefer regional anaesthesia to general anaesthesia (*P* < .05). It was also found in the survey that those who preferred regional anaesthesia to general anaesthesia were significantly more concerned about COVID-19 transmission rather than anaesthesia/surgical anxiety (*P* < .01) (Table 2).

It was also investigated in the study whether there were significant differences between the distribution rates of the primary concern causes according to educational status, vaccination status, occupation, preferred anaesthesia type, and caesarean number. The results show that there were no significant differences in terms of educational status, vaccination status, occupation, and caesarean number variables (*P* > .05). However, it was also found that the rates of primary concern cause differed according to the type of preferred anaesthesia (*P* < .01) (Table 3).

When the relationship between the anxiety levels of the patients and their anaesthesia preferences was evaluated, it was found that the mean surgical anxiety scores of the patients whose primary concern was surgery were significantly higher, and the mean Beck Anxiety scores of those who preferred general anaesthesia were higher (*P* < .05). However, no significant differences were detected between the groups in terms of other variables (*P* > .05) (Table 4).

The results obtained with the correlation with the Beck Anxiety, APAIS, sub-dimensions of APAIS, anaesthesia anxiety, and surgical anxiety and information obtained show that there were moderate-high and positive significant relationship between all variables (*P* < .001). Also, the Cronbach alpha reliability coefficients ranged between 0.74 and 0.93 (Table 5).

The lowest and highest values in Beck Anxiety, APAIS, sub-dimensions of anaesthesia anxiety, surgical anxiety, and mean standard deviation values in obtaining information dimensions are given in Table 6.

## Discussion

It was determined that 50.7% (71/140) of the pregnant women who preferred general anaesthesia in their previous caesarean sections preferred regional anaesthesia during the COVID-19 pandemic. It was also determined that patients who had primary anxiety because of COVID-19 contamination anxiety during hospitalization and caesarean section preferred regional anaesthesia more (36/55), and the primary reason for anxiety in the majority of patients who preferred regional anaesthesia was COVID-19 contamination anxiety (36/71).

Elective surgical procedures were postponed in many countries after COVID-19 became a pandemic problem to limit the spread of infection and to conserve healthcare resources, including the staff, surgery rooms, and anaesthesia devices, which resulted in a significant reduction in surgical admissions worldwide.^12^ Regional anaesthesia became the preferred option because it can provide an alternative safe anaesthetic care plan by eliminating the requirement for aerosol-generating procedures in patients who undergo surgery. Although aerosol-generating interventions such as mask ventilation, tracheal intubation, non-invasive ventilation, and tracheal aspiration in general anaesthesia in the pandemic period increase the risk of infection, the fact that these risks are lower with regional anaesthesia^13,14^ has made regional anaesthesia preferable during the pandemic process.^15^

In the present study, it was found that 50.7% (71/140) of the patients, who were interviewed regarding the anaesthesia for caesarean section and who preferred general anaesthesia voluntarily without any contraindications in their previous caesarean sections, changed their preferences during the pandemic period and preferred regional anaesthesia. Although the change in anaesthesia preferences of the patients in our study can be associated with their previous experiences, the poor experiences of previous surgery and anaesthesia were not questioned in the study. However, anaesthesia-related anxiety is higher in patients who choose regional anaesthesia than in other studies. It is considered that this develops because of the risk of transmission in the pandemic. A study conducted by Thorp et al^16^ showed that previous experiences did not reduce preoperative anxiety. Since all the patients in the patient group had undergone previous surgery in the present study, the effectiveness of this in the study results was not targeted.

Although no significant differences were detected between the general anaesthesia and regional anaesthesia preferences of those who claimed anaesthesia anxiety and surgical anxiety as the primary cause of anxiety, it was found that those who claimed COVID-19 contamination anxiety as the primary concern preferred regional anaesthesia at significantly higher levels. The primary concern of patients who preferred regional anaesthesia was the concern of COVID-19 infection. Various factors such as the increased anxiety and depression inherence in pregnancy, surgical anxiety, previous anaesthesia experience, and lack of knowledge on anaesthesia play roles in anaesthesia preferences of patients. The anaesthesia applied for the procedure is perceived as a danger by patients as well as the surgical procedure itself, and this perception leads to anxiety.^17-19^ Also, the fear of being infected with COVID-19 is now an additional reason for anxiety.

People can be expected to show symptoms of anxiety and hopelessness during quarantine periods. The COVID-19 pandemic is an additional risk factor that likely increases stress in pregnant women who are already prone to depression and anxiety. These women may naturally be concerned not only for their own health but also for their unborn babies because of this contagious disease. Pregnancy is a particularly vulnerable period, and studies conducted on mental health measures report that pregnant women face higher levels of anxiety during an infectious disease outbreak.^20^ During pregnancy, the increased level of progesterone increases the incidence of depression in addition to the physiological changes. For this reason, the anaesthesia preferences of the patient group who prefer elective caesarean section may differ when compared to other patients.

Hospital and surgery room employees can be asymptomatically infected people. The symptoms may appear 2-14 days after the exposure to COVID-19, and the incubation period ranges from 4 to 7 days during which any infected patient may be asymptomatic and contagious.^21^ For this reason, there may be a risk of infection transmission during intubation, extubation, and ventilation stages in patients who undergo general anaesthesia, despite all the care and precautions both for the patient and the newborn baby. Therefore, the fact that pregnant women prefer regional techniques in which the upper respiratory tract is not used during anaesthesia protects them and healthcare employees against the risk of COVID-19 transmission.

Although the surgical anxiety score of the APAIS subgroups of the patients who showed their surgical anxiety as the primary cause of anxiety was higher at significant levels, the Beck Anxiety score of the patients who preferred general anaesthesia was significant. It was an expected result that the surgical anxiety of patients whose primary concern was surgery was found to be high. The significantly higher Beck Anxiety scores, which is a general anxiety scale, in patients who preferred general anaesthesia can be explained by the anxiety of patients with anxiety regarding staying awake during surgery. Previous studies^5,22^ reported that the anxiety of staying awake during surgery was one of the most common concerns for choosing general anaesthesia.^6^

According to the findings of the present study, no significant differences were detected between education and anxiety levels. It is reported in the literature that high-level anxiety is compatible with educated patients as they are aware of complications.^3,4,23,24^ On the other hand, there are conflicting studies regarding the relationship between education and anxiety.^25,26^ No significant differences were detected between age and anxiety levels, which is another demographic data. This finding was consistent with the results reported in the study by Domar et al.^23^ The number of previous caesarean sections did not cause statistically significant differences between the groups in terms of anxiety levels. Similarly, although the number of pregnant women who received the COVID-19 vaccine was low, no significant differences were detected between the groups with and without the vaccine.

The limitation of the study is that the patients included in our study were not questioned about their previous surgical and anaesthesia experiences.

## Conclusion

Pregnancy is a difficult process with its physical and psychological problems. Pregnancy during the pandemic causes more hardships for pregnant women psychologically and may even create more anxiety for their babies and themselves, especially with the fear of contagious disease regarding the process in the hospital for caesarean section. It was found that more than half of the pregnant women who preferred general anaesthesia based on their own preferences in previous caesarean sections changed their preferences and preferred regional anaesthesia in caesarean sections during the pandemic. We believe that the preoperative information provided on COVID-19 is effective in the choice of regional anaesthesia, which is considered to have a low risk of transmission. For this reason, there is a need for additional studies to be conducted on the effects of providing information about the preoperative pandemic on the choice of anaesthesia.

We think that it is important to inform patients about the pandemic precautions in the surgery room and hospital during the pandemic, as well as routine information given at the preoperative anaesthesia appointment. We believe that more comprehensive studies must be conducted on anaesthesia preferences and primary causes of anxiety during the pandemic period.

## Figures and Tables

**Table 1.** Patient Characteristics

Patient Characteristics		n	%
Age group	18-24	17	12.1
	25-34	90	64.3
	35+	33	23.6
Caesarean number	1	81	57.9
	2	42	30.0
	3	17	12.1
Education	Primary school	20	14.3
	Secondary school	44	31.4
	High school	33	23.6
	University	43	30.7
Vaccination status	Vaccinated	19	13.6
	Not vaccinated	121	86.4
Occupation	Housewife	108	77.1
	Employee	20	14.3
	Civil servant	9	6.4
	Healthcare employee	3	2.1
Preferred Anaesthesia	General	69	49.3
	Regional	71	50.7
Primary concern cause	Anaesthesia	48	34.3
	Surgery	37	26.4
	COVID-19 infection concern	55	39.3

**Table 2.** The Distribution Rates of Participants Who Had Anxiety due to COVID-19 Contagion According to Preferred Anaesthesia and Primary Cause of Anxiety of Participants Who Preferred Regional Anaesthesia

Variables		Observed, N	Expected, N	*χ*²
Preferred anaesthesia^*^	General	19	27.5	5.255 *P* = .022
	Regional	36	27.5	
	Total	55	-	
Primary concern cause^**^	Anaesthesia	17	23.7	9.662 *P* = .008
	Surgery	18	23.7	
	COVID-19 infection	36	23.7	
	Total	71	-	

^*^The sampling consisted of participants with COVID-19 infection concerns;

^**^The sampling consisted of participants who preferred regional anaesthesia.

**Table 3.** The Distribution Rates according to Primary Concern Cause, Educational status, Age group, Vaccination status, Occupation, and Preferred Anesthesia Type

Variables		Anesthesia Concern	Surgery Concern	Covid-19 Infection Concern	Total	χ²
Educational status						
Primary school	n	6	5	9	20	4.758 *P* = .575
	%	12.5	13.5	16.4	14.3	
Secondary school	n	15	9	20	44	
	%	31.3	24.3	36.4	31.4	
High school	n	14	11	8	33	
	%	29.2	29.7	14.5	23.6	
University	n	13	12	18	43	
	%	27.1	32.4	32.7	30.7	
Age group						
18-24	n	6	4	7	17	2.677 *P* = .613
	%	12.5	10.8	12.7	12.1	
25-34	n	27	26	37	90	
	%	56.3	70.3	67.3	64.3	
35+	n	15	7	11	33	
	%	31.3	18.9	20.0	23.6	
Vaccination status						
Vaccinated	n	5	8	6	19	2.784 *P* =.249
	%	10.4	21.6	10.9	13.6	
Not vaccinated	n	43	29	49	121	
	%	89.6	78.4	89.1	86.4	
Occupation						
Housewife	n	36	29	43	108	2.338 *P* =.886
	%	75.0	78.4	78.2	77.1	
Employee	n	7	5	8	20	
	%	14.6	13.5	14.5	14.3	
Civil servant	n	3	2	4	9	
	%	6.3	5.4	7.3	6.4	
Healthcare Employee Civil servant	n	2	1	0	3	
	%	4.2	2.7	.0	2.1	
Preferred Anesthesia						
General	n	31	19	19	69	9.338 *P* **=.009**
	%	64.6	51.4	34.5	49.3	
Regional	n	17	18	36	71	
	%	35.4	48.6	65.5	50.7	
Cesarean Number						
1	n	25	22	34	81	4.955 *P* =.252
	%	52.1	59.5	61.8	57.9	
2	n	19	8	15	42	
	%	39.6	21.6	27.3	30.0	
3	n	4	7	6	17	
	%	8.3	18.9	10.9	12.1	
Total	n	48	37	55	140	
	%	100.0	100.0	100.0	100.0	

**Table 4.** Comparison of Anaesthesia Anxiety, Surgical Anxiety, Receiving Information, and Beck Anxiety Averages According to Age Groups, Education Levels, Primary Anxiety Reasons, Preferred Anaesthesia Types, Vaccination Status, and Number of Caesarean Sections

		n	Anaesthesia Anxiety		Surgery Anxiety		Receiving Information		Beck Anxiety	
			Mean ± SD	*t/F*	Mean ± SD	*t/F*	Mean ± SD	*t/F*	Mean ± SD	*t/F*
Age group	18-24	17	3.88 ± 1.27	*F* = 1.081 *P* = .342	4.47 ± 2.76	*F* = .402 *P* = .670	5.24 ± 2.95	*F* = 1.563 *P* = .213	31.59 ± 7.02	*F* = .414 *P* = .662
	25-34	90	4.63 ± 1.96		4.99 ± 2.15		5.7 ± 2.46		34.27 ± 11.39	
	35+	33	4.45 ± 2.15		4.79 ± 2.37		4.82 ± 2.36		34 ± 12.19	
Educational status	Primary school	20	4.05 ± 1.61	*F* = 1.087 *P* = .357	4.35 ± 1.95	*F* = 2.266 *P* = .084	5.3 ± 2.56	*F* = .758 *P* = .519	30 ± 6.51	*F* = 1.276 *P* = .285
	Secondary school	44	4.43 ± 1.95		4.55 ± 2.19		5.41 ± 2.73		34.23 ± 11.58	
	High school	33	4.33 ± 2.07		4.7 ± 2.56		5 ± 2.47		36.09 ± 11.96	
	University	43	4.91 ± 1.97		5.6 ± 2.16		5.86 ± 2.29		33.63 ± 11.51	
Primary concern cause	Anaesthesia	48	4.65 ± 2.03	*F* =.284 *P* = .753	4.6 ± 2.25	4.190 *P* =.017	5.48 ± 2.74	*F* = 2.347 *P* = .099	33.56 ± 10.94	*F* = .042 *P* = .959
	Surgery	37	4.32 ± 1.87		5.78 ± 2.44		4.73 ± 2.27		34.27 ± 12.12	
	Covid infection suspicion	55	4.49 ± 1.94		4.51 ± 2.04		5.87 ± 2.4		33.89 ± 10.77	
Preferred Anaesthesia Type	General	69	4.77 ± 2.09	*t* = 1.619 *P* = .108	5 ± 2.37	*t* = .621 *P* =.535	5.49 ± 2.55	*t *= .264 *P* = .792	35.94 ± 12.22	*t *= 2.194 *P* = .030
	Regional	71	4.24 ± 1.76		4.76 ± 2.19		5.38 ± 2.49		31.87 ± 9.6	
Vaccination status	Vaccinated	19	4.68 ± 2.29	*t* = .443 *P* = .658	5.42 ± 1.77	*t* = 1.119 *P* =.265	6.05 ± 2.15	*t* = 1.153 *P* = .251	35.84 ± 11.3	*t* = .827 *P* = .410
	Not vaccinated	121	4.47 ± 1.89		4.79 ± 2.34		5.34 ± 2.56		33.57 ± 11.11	
Caesarean nNumber	1	81	4.40 ± 1.90	*F* = .293 *P* = .747	4.94 ± 2.32	*F* =.646 *P* =.526	5.69 ± 2.59	*F* =1.473 *P* = .233	33.98 ± 10.6	*F* = .192 *P *= .825
	2	42	4.69 ± 2.08		5.00 ± 1.98		5.29 ± 2.43		34.31 ± 11.6	
	3	17	4.47 ± 1.19		4.29 ± 2.58		4.59 ± 2.24		32.35 ± 12.5	

SD, standard deviation.

**Table 5.** Relations Between Beck Anxiety Inventory, Amsterdam Preoperative Anxiety and Information Scale, and Sub-Dimensions of Amsterdam Preoperative Anxiety and Information Scale

Variables	1	2	3	4	Cronbach α
1. Beck anxiety	1				.93
2. Anaesthesia anxiety	.498^**^	1			.74
3. Surgery anxiety	.439^**^	.621^**^	1		.80
4. Receiving information	.372^**^	.464^**^	.460^**^	1	.83
5. APAIS	.517^**^	.883^**^	.916^**^	.513^**^	.84

**P < .001.

**Table 6.** Mean, Standard Deviation, and Lowest-Highest Values for Study Variables

Variables	Minimum	Maximum	Mean	Standard Deviation
Beck anxiety	21.00	67.00	33.88	11.12
APAIS	4.00	20.00	9.38	3.80
Anaesthesia anxiety	2.00	10.00	4.50	1.94
Surgery anxiety	2.00	10.00	4.88	2.27
Receiving information	2.00	10.00	5.44	2.51
